# Is molecular testing necessary for squamous cell carcinoma?

**DOI:** 10.1093/oncolo/oyaf226

**Published:** 2025-08-13

**Authors:** Tadashi Nishimura, Hajime Fujimoto

**Affiliations:** Department of Pulmonary Medicine, Mie Chuo Medical Center, Tsu City, Mie 514-1101, Japan; Department of Pulmonary and Critical Care Medicine, Mie University Graduate School of Medicine, Tsu, Mie 514-8507, Japan

To the editor

Last year, the overall survival update of the CROWN study and the results of the MARIPOSA study were published.^1^^,^^2^ These data reminded us of the usefulness of targeted therapies and we have an urgent need for molecular testing of non-small cell lung cancer. Recently, a great review of molecular testing by Drs. Frédérique Penault-Llorca and Mark A. Socinski has been published.^3^ As the review focuses on non-small cell lung cancer, it also includes squamous cell carcinoma. Since I experienced a case of the epidermal growth factor receptor-mutated lung squamous cell carcinoma that did not respond to osimertinib,^4^ I have concerns about the need for molecular testing and effectiveness of targeted therapy for squamous cell carcinoma. The evidence for molecular testing in squamous cell carcinoma is scarce and its development is not easy.

The guidelines cited by the authors state that all histological subtypes including squamous cell carcinoma should undergo molecular testing.^3^ Alternatively, in the case of squamous cell carcinoma, testing is recommended for younger patients, light smokers, or long-term ex-smokers.^3^ Recently, it has been reported that genetic mutations are more common in squamous cell carcinoma patients who have no history of smoking.^5^ However, squamous cell carcinoma without a history of smoking is rare, accounting for only 2% of those surveyed, and there is no statistically significant difference in the efficacy of the treatment. Even if molecular testing is performed in patients without a history of smoking, it is unclear whether targeted therapy is effective.

The Lung-MAP SWOG S1400 trial is a large study of the efficacy of targeted therapy for squamous cell carcinoma.^6^ This trial showed that targeted therapies are not effective for second-line treatment of squamous cell carcinoma.^6^ Although subsequent clinical trials of some targeted therapies have included patients with squamous cell carcinoma as non-small cell lung cancer, the number of squamous cell carcinoma patients in these clinical trials is small.^7^ The effectiveness of the targeted therapies must be further evaluated through the accumulation of evidence for each drug. However, there is a possibility of publication bias in case reports, with only those reporting positive outcomes being published.^8^ Even if a systematic review is conducted, reliability is low, and clinical trials are necessary for each targeted therapy. However, conducting clinical trials for each treatment modality requires a significant amount of time.

It is still unclear whether molecular testing of squamous cell carcinoma is necessary and whether targeted therapies are effective. More evidence is needed to address these concerns, including large clinical trials or platform trials evaluating the effectiveness of targeted therapies for squamous cell carcinoma.
